# Is Autophagy a Friend or Foe in SARS-CoV-2 Infection?

**DOI:** 10.3390/v16091491

**Published:** 2024-09-20

**Authors:** Asifa Khan, Jiaxin Ling, Jinlin Li

**Affiliations:** 1 Department of Medical Biochemistry and Microbiology, The Biomedical Center, Uppsala University, P.O. Box 582, 751 23 Uppsala, Sweden; 2 Biochemistry Unit, Department of Molecular Medicine, University of Pavia, 27100 Pavia, Italy; 3 Zoonosis Science Center, Uppsala University, 751 23 Uppsala, Sweden

**Keywords:** SARS-CoV-2, autophagy, virophagy, incomplete autophagy, autophagy modulator, antivirals

## Abstract

As obligate parasites, viruses need to hijack resources from infected cells to complete their lifecycle. The interaction between the virus and host determines the viral infection process, including viral propagation and the disease’s outcome. Understanding the interaction between the virus and host factors is a basis for unraveling the intricate biological processes in the infected cells and thereby developing more efficient and targeted antivirals. Among the various fundamental virus–host interactions, autophagy plays vital and also complicated roles by directly engaging in the viral lifecycle and functioning as an anti- and/or pro-viral factor. Autophagy thus becomes a promising target against virus infection. Since the COVID-19 pandemic, there has been an accumulation of studies aiming to investigate the roles of autophagy in SARS-CoV-2 infection by using different models and from distinct angles, providing valuable information for systematically and comprehensively dissecting the interplay between autophagy and SARS-CoV-2. In this review, we summarize the advancements in the studies of the interaction between SARS-CoV-2 and autophagy, as well as detailed molecular mechanisms. We also update the current knowledge on the pharmacological strategies used to suppress SARS-CoV-2 replication through remodeling autophagy. These extensive studies on SARS-CoV-2 and autophagy can advance our understanding of virus–autophagy interaction and provide insights into developing efficient antiviral therapeutics by regulating autophagy.

## 1. Introduction

Severe acute respiratory syndrome coronavirus 2 (SARS-CoV-2), which emerged in late 2019, is the causative agent of the COVID-19 pandemic. The virus circulated globally and acquired various mutated forms, referred to as variants of concern (VOC). These variants include Alpha, Beta, Gamma, Delta, Omicron, and so on [1]. Since the outbreak in January 2020, more than 700 million cases have been reported, with over 7 million deaths worldwide [2]. Although WHO declared an end to the COVID-19 pandemic as a global health emergency on 5 May 2023 [3], SARS-CoV-2 is still circulating worldwide and poses a continuous threat to public health. The COVID-19 pandemic is unlikely to be the last one caused by viruses.

The COVID-19 pandemic encouraged intensive studies on the interactions between viruses and hosts, which is the basis for a deeper understanding of how viruses are able to successfully replicate in the host cell and the development of effective antiviral treatments. Among various important biological processes that engage in viral infection, autophagy plays intricate roles at the distinct stages of virus infection. Autophagy is a catabolic pathway involved in breaking down cellular contents via lysosomes. Basal autophagy regulates cell homeostasis by controlling protein turnover and removing damaged or surplus organelles [4]. Autophagy induced by stress provides a means of adaptation and cell survival by minimizing cell growth activities and recycling nutrients from degraded cellular materials [5]. Depending on the stimuli and downstream signaling, autophagy can also induce non-apoptotic cell death [6]. Moreover, on the one hand, autophagy serves as an innate immune response against viruses and other pathogens [7]. On the other hand, the ever-evolving virus has used multiple strategies to antagonize or hijack cellular autophagy, by which to facilitate viral replication. In this review, we summarize the intricate interactions between SARS-CoV-2 infection and autophagic machinery, with highlights on the recent advancements in the studies, providing insights into understanding the interplay between virus and autophagy.

### 1.1. SARS-CoV-2

SARS-CoV-2, belonging to the *Coronaviridae* family, is a positive-sense single-stranded RNA virus with the largest genome among RNA viruses, encoding 29 viral proteins [8], whereas the translation of some additional ORFs into functional proteins is predicted by computational approaches [9,10]. ORF1ab, the largest gene of SARS-CoV-2, is responsible for encoding 16 non-structural proteins (NSP1-16), which play vital roles in viral replication and forming resistance to host immune responses. One-third of the genome consists of regions for four structural proteins—spike (S), envelope (E), membrane (M), nucleocapsid (N)—and overlapping frames for accessory proteins, including ORF3a, ORF6, ORF7a, ORF7b, ORF8, ORF9b, and ORF10. The structural proteins form the virion, and the accessory proteins confer virulence by manipulating the host’s cellular pathways.

### 1.2. Key Players in Autophagic Machinery

**Figure 1:**
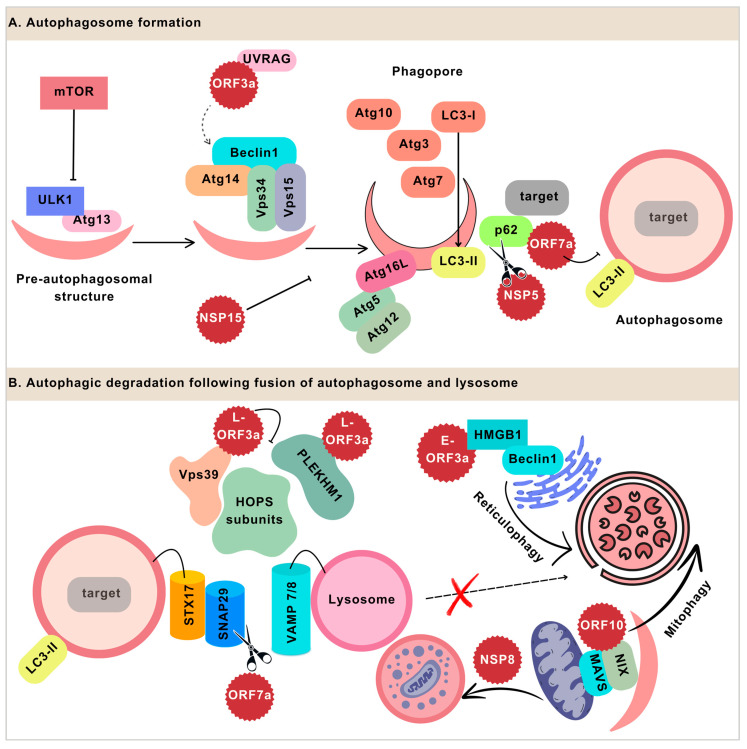
SARS-CoV-2 proteins hijacking autophagic machinery to affect the formation and maturation of autophagosomes. (**A**). Autophagy vesicle formation initiates ULK1 and Atg13 autophagy-related proteins. mTOR is an inhibitor of the initiation step. The vesicle nucleation involves the recruitment of other proteins including Beclin1, which forms a complex with Atg14, Vps34, and Vps15. The interaction of the SARS-CoV-2 protein ORF3a with UVRAG may drive the process towards autophagosome formation and prevent the autophagosome’s maturation by hindering the interaction of UVRAG with Beclin1. Other autophagy-related proteins such as Atg10, Atg7, and Atg3 catalyze the two conjugation reactions to form the Atg5–Atg12–Atg16L complex and LC3-II, which leads to the enclosure of autophagosome vesicles. The SARS-CoV-2 protein NSP15 inhibits autophagosome formation; the mechanism of this process and the phase of vesicles at which it is involved are not yet determined. The adaptor protein, p62, facilitates the interaction of the target molecules with LC3-II, which brings the target closer to the autophagosome for engulfment. SARS-CoV-2 NSP5 cleaves p62, inhibiting autophagic degradation. ORF7a is also known to interact with p62; this interaction of ORF7a prevents the fusion of the autophagosome with the lysosome. (**B**). ORF7a prevents the fusion by degrading SNAP29 through the caspase pathway. The degradation of SNAP29 compromises the interaction with the receptor on the lysosome, vesicle-associated membrane protein 7/8 (VAMP7/8). The ORF3a localized to the lysosome (L-ORF3) binds to Vps39, a subunit of the HOPS complex. The interaction with ORF3a prevents the HOPS formation which is required for mediating the tethering of the autophagosome on the lysosome. ORF3a also disrupts HOPS formation by binding to another HOPS subunit, PLEKHM1. While autophagic flux is reduced due to these viral proteins, the selective degradation of the host ER and mitochondrial proteins is facilitated by some SARS-CoV-2 proteins. The ER-localized ORF3a (E-ORF3a) promotes reticulophagy. ORF3a interacts with high mobility group box 1 protein (HMGB1), which is a damage-associated molecular pattern. By increasing the interaction of HMGB1 with Beclin1, ORF3a promotes reticulophagy of the ER. ORF10 of SARS-CoV-2 targets mitochondrial antiviral signaling (MAVS) for autophagy through autophagy receptor NIX. The NSP8 protein of SARS-CoV-2 also targets mitochondria, but the autophagy is incomplete, which leads to the accumulation of autophagosomes.

Macro-autophagy, micro-autophagy, and chaperone-mediated autophagy are three established pathways of autophagy [11], and macro-autophagy is the most common mode of autophagy. The reviewed studies in this article focused on macro-autophagy, referred to as ‘autophagy’ from here on. In this pathway, the target material is engulfed by a double-membrane vesicle called an autophagosome. The membranes that form autophagosomes originate from the endoplasmic reticulum (ER), mitochondria, Golgi apparatus, ER-Golgi intermediate compartment (ERGIC), or plasma membrane [12]. Several proteins are involved in the formation and maturation of autophagosomes (Figure 1). Beclin1/Atg6 (autophagy-related gene 6) is one of the key components in the formation of the initial membranous structure, the phagopore [13]. The phagopore is an unclosed vesicle surrounding the target of the autophagy. Beclin1 interacts with vesicular protein sorting 34 (Vps34), which plays a role in fetching other Atg proteins and phagopore elongation [14]. Other proteins include Atg14 and Vps15, constituting the complex Beclin1–Atg14–Vps15–Vps34. Subsequently, other Atg proteins including Atg7, Atg10, and Atg3 drive the two types of conjugation reactions to form the Atg5–Atg12–Atg16L complex and LC3-II [14]. LC3-II is the lipidated form of LC3-I, which is cleaved from the Atg8 family protein, microtubule-associated proteins 1A/1B light chain 3B (LC3B). The Atg5–Atg12–Atg16L complex dissociates from the complete autophagosome, whereas LC3-II remains attached. Hence, LC3-II is a prominent biomarker of autophagosome formation. The adaptor proteins facilitate the interaction of ubiquitinated targets with LC3-II [15], bringing the targets into the autophagosome. The autophagy receptors, including LC3-II, are later degraded along with the target cargo following the fusion with the lysosome. Therefore, the elevated levels of LC3-II or adapter proteins either indicate compromised downstream degradation or increased upstream autophagosome formation.

The mature autophagosome carrying the cargo fuses with the lysosome through tethering receptors. The homotypic fusion and protein sorting (HOPS) [16] complex, comprising Vps11, Vps16, Vps33A, Vps39, and Vps41, is recruited by autophagosomes and mediates the docking of the autophagosome on the lysosome [17]. The HOPS complex facilitates the interaction of adaptor proteins syntaxin-17 (STX17) and synaptosome-associated protein 29 (SNAP29) on the autophagosome with the receptor protein vesicle-associated membrane protein 8 (VAMP8) on the lysosome [17]. The resulting trans-SNARE (SNAP-Receptor) complex derives the fusion of the membranes to form autolysosomes. Some studies also report the role of the Beclin1 complex (Beclin1-UVRAG-Vps15-Vps34-Rubicon) with UV radiation resistance-associated gene protein (UVRAG) in the fusion of the autophagosome and lysosome [18]. The acidic environment and hydrolases of lysosomes break down the cargo, releasing the nutrients or pathogen fragments to stimulate the immune response [7,19].

### 1.3. The Interplay between Autophagy and Viruses

The role of autophagy in virus infection is pertinent, as autophagy is one of the important components of innate immune responses during host defense against pathogens invading the host cells. In response to the selective autophagy that eliminates viruses, namely “virophagy” [20,21], viruses have evolved mechanisms to evade autophagic degradation. Several viruses alter the autophagic activity of the cell, either as a cellular stress response upon infection or to exploit the autophagy machinery for their replication. Among these, the viruses belonging to the family *Coronaviridae* have long been known to utilize double-membrane vesicles (DMVs) for their replication [22,23]. Furthermore, Zika virus (ZIKV) induces autophagy initiation to foster its replication [24]. Human parainfluenza virus type 3 (HPIV3) [25], influenza A virus (IAV) [26], and Epstein–Barr virus (EBV) [27] prevent the fusion of autophagosomes with lysosomes to escape from lysosomal degradation. On the other hand, human cytomegalovirus (HCMV) [28,29] and herpes simplex virus type 1 (HSV-1) [30] decrease autophagic degradation by blocking autophagy initiation. Moreover, viruses also induce the selective autophagy of mitochondria [31] to decrease the interferon (IFN) antiviral response. Thus, viruses possess both pro-autophagic and anti-autophagic properties to hijack the DMVs for their replication or to evade innate immunity, highlighting a complex interplay between viruses and autophagy.

## 2. Modulation of Autophagy by SARS-CoV-2

### 2.1. SARS-CoV-2 Infection Modulates the Autophagy-Related Proteins

The critical roles of autophagy in virus infection and associated diseases draw much attention to the interaction of SARS-CoV-2 and host autophagy. In order to pinpoint the effects of SARS-CoV-2 infection on autophagy and elucidate if dysregulated autophagy is associated with the outcome of COVID-19, autophagy-associated components were studied in COVID-19 patients [32,33]. These components include Beclin1 and other autophagy biomarkers, including LC3-II and p62. Beclin1 levels were significantly higher in 108 COVID-19 patients (70 with mild cases and 38 with severe cases) compared to 21 healthy controls [32]. Similarly, the amount of Beclin1 was considerably elevated in the group of severe cases than in the mild cases. Furthermore, there was a positive correlation between the Beclin1 levels and hematological markers, including the blood coagulation markers D-dimer and fibrinogen, inflammation markers such as the erythrocyte sedimentation rate (ESR) and C-reactive protein (CRP), and biochemical indicators of liver/kidney damage including alanine aminotransferase (ALT), aspartate aminotransferase (AST), creatinine, and blood urea nitrogen (BUN). The findings suggest that an increased Beclin1 level is linked with COVID-19, and Beclin1 is potentially associated with disease severity. The increase in Beclin1 level in COVID-19 patients, however, does not provide direct evidence of increased autophagy, as Beclin1 is a multifunctional protein [34]. When Beclin1 interacts with an anti-apoptotic protein, BCL2, it suppresses autophagosome formation [35]. Hence, post-translational modifications and interactions of autophagy-related proteins with other proteins are important to determine the potential effect on autophagy. One of the examples of such post-translational modifications is citrullination. Citrullination involves the conversion of positively charged arginine residues in proteins to citrulline, resulting in no net charge. Consequently, citrullination may trigger autophagy in response to unfolded proteins. The relationship between citrullination and autophagy levels has previously been studied in systemic lupus erythematosus [36]. Interestingly, the unfolded protein response triggered by the SARS-CoV-2 ORF3a protein and its connection to increased autophagosome formation has also been observed [37], which is discussed in the next section of this review.

The phosphorylation levels of autophagy-related proteins were evaluated in response to SARS-CoV-2 infection in vitro [38]. AMPK and mTOR are two key regulators of autophagy in energy depletion and starvation [39]. AMPK-dependent phosphorylation of the autophagy initiation protein Unc-51-like kinase 1 (ULK1) promotes autophagy [39], while mTOR-dependent phosphorylation of ULK1 inhibits the autophagy-related function of ULK1 [39]. The mTORC1-dependent inhibitory phosphorylation of ULK1 did not change significantly in response to SARS-CoV-2 infection [38]. However, AMPK-dependent activating phosphorylation of ULK1 was significantly decreased in the SARS-CoV-2-infected cells. Corresponding to the decreased ULK1 activation, ULK1-dependent phosphorylation of Beclin1 (pBECN1^S15^) and Atg14 (pATG14^S29^) was also downregulated. Based on these findings, Gassen et al. [38] proposed the reduced pATG14^S29^ level to be a potential mechanism of SARS-CoV-2-induced incomplete autophagy.

Intriguingly, the levels of LC3-II and p62 were elevated in SARS-CoV-2-infected cells, which is consistent in many studies [40,41,42,43,44,45,46,47]. These findings suggest the compromised degradation activity of autophagy. Furthermore, the evidence from fluorescence microscopy assays showed a decreased number of autolysosomes in SARS-CoV-2-infected cells [38] and an accumulation of autophagosomes in SARS-CoV-2 protein-transfected cells [41,48,49]. Electron microscopy also showed increased autophagosome accumulation in infected/transfected cells [42,43,48,49]. Altogether, these findings indicate impaired fusion of autophagosomes with lysosomes, thereby resulting in incomplete autophagy, characterized by the formation of autophagosomes but not resulting in lysosomal degradation. In addition to cell lines, SARS-CoV-2 also increased LC3-II levels in the lung tissue of an in vivo model of *Macaca fascicularis* macaques [43]. The levels of LC3-II and p62 in peripheral blood mononuclear cells (PBMCs) of COVID-19 patients were significantly upregulated compared to healthy controls [33]. In contrast, another study presented a decreased conversion of LC3-I to LC3-II in PBMCs of COVID-19 patients [50]. However, it is worth noting that assessing the conversion rate of LC3-I to LC3-II and measuring the total levels of LC3-II along with p62 are slightly different ways of assessing autophagy [51] (pp. 53–59, p. 82). While the decreased ratio of LC3-II to LC3-I might indicate either decreased lipidation of LC3-I or rapid lysosomal degradation of LC3-II, the increased levels of LC3-II along with elevated p62 indicate the blockage of autophagic flux due to decreased autophagolysosome content degradation. Overall, the accumulation of LC3-II with SARS-CoV-2 infection [33,43] suggests a blockage in autophagic degradation.

Some computational and transcriptomics studies also provide evidence of SARS-CoV-2-mediated autophagy perturbations [45,52,53]. scRNAseq analysis of epithelial cells isolated from the bronchoalveolar lavage fluid (BALF) of COVID-19 patients and matched controls exhibited the downregulation of autophagy-related genes [45]. Moreover, the significant differentially expressed mRNA and miRNA in COVID-19 patients compared to matching controls coincide with the mRNA and miRNA reported in the Human Autophagy Database (http://autophagy.lu, accessed on 18 September 2024), which shows that the differentially expressed mRNA and miRNA in COVID-19 patients are related to autophagy [52]. The downregulation of autophagy-related genes was also observed in SARS-CoV-2-infected human nasopharyngeal samples and A549 cells [53].

Likewise, dsRNA of SARS-CoV-2 shows a tendency to impair mitophagy, the selective autophagy of mitochondria [54]. In SARS-CoV-2-infected cells, the expression of serine/threonine-protein kinase (Pink1) and parkin was upregulated. Pink1 and parkin regulate the ubiquitination of damaged mitochondria, and in SARS-CoV-2-infected cells, the colocalization of Hsp60 with Pink1 and Parkin was increased. SARS-CoV-2 also promoted the colocalization of p62 and mitochondria. These observations indicate the activation of SARS-CoV-2-induced mitophagy. SARS-CoV-2 dsRNA colocalized with mitochondrial markers, heat shock protein 60 (Hsp60), and mitochondrial import receptor subunit (Tom20), which suggests that dsRNA could be the trigger for mitophagy activation. The colocalization of p62 with the mitochondrial marker Hsp60 was reduced when Pink1 was knocked down [54]. Hence, SARS-CoV-2 might induce mitophagy by upregulating the Pink1/parkin pathway. While the activation of mitophagy was triggered by SARS-CoV-2, it did not lead to mitochondrial clearance as the levels of Hsp60 were sustained. Thus, the mitophagy was incomplete. SARS-CoV-2 hinders the interaction of p62 with LC3-II, which is the proposed mechanism by which SARS-CoV-2 compromises the mitophagy [54]. The Hsp60 did not exhibit colocalization with LC3 or the lysosomal marker Lamp2b in infected cells. However, in another study, the colocalization of GFP-LC3 and a DsRed-tagged mitochondrial marker protein, Mito, was observed in the SARS-CoV-2-infected HeLa-ACE2 cells [55], which may favor mitophagy. While mitochondrial clearance is compromised with SARS-CoV-2 infection [54], the selective degradation of mitochondrial proteins involved in host innate immunity is governed by the viral protein ORF10 [55], which is discussed in a later section of this article.

The targeted autophagy of mitochondria by SARS-CoV-2 might be a time-dependent phenomenon. Sui et al. [56] demonstrated how SARS-CoV-2 infection affects the level of p62 and TANK-binding kinase 1 (TBK1), a signaling molecule in the IFN-β production pathway, at different time points. SARS-CoV-2-infected Calu-3 cells demonstrated decreased levels of TBK1 and p62 at 8 h post infection. The degradation of TBK1 may be associated with SARS-CoV-2-mediated autophagy, as indicated by the reduced level of p62. However, the levels of TBK1 and p62 were recovered at 16 h post infection. The study [56] deduced that at different time points, viral proteins such as ORF3a might impair autophagy, which corresponds to the restored levels of TBK1 and p62. Hence, the interaction of SARS-CoV-2 with autophagy machinery is complex and time-dependent.

### 2.2. The Molecular Mechanisms by Which SARS-CoV-2 Remodels Autophagy

To further explore the mechanisms of SARS-CoV-2-induced changes in autophagy, the SARS-CoV-2 proteins were screened to evaluate their potential to affect autophagic flux [41,48,49,57]. The levels of LC3-II in cells increased upon transfection with plasmids expressing the SARS-CoV-2 proteins ORF3a [37,41,48,49,58], ORF7a [42,48,49], membrane protein-M [49,59], NSP6 [49,60], NSP8 [47], E [59], and S [46]. The increase in LC3-II was accompanied by increased p62 levels upon transfection with ORF3a [37,41,48,49,61], ORF7a [42], and NSP8 [47], indicating incomplete autophagy induction. ORF3a and ORF7a impair the fusion of autophagosomes and lysosomes, which results in incomplete autophagy. The fluorescence microscopy assays in multiple studies indicate increased autophagosome levels and decreased autolysosome levels in the ORF3a- [37,48,49] and ORF7a [42]-expressing cells. Moreover, the colocalization of ORF3a [48,49] with the lysosomal biomarker lysosomal-associated membrane protein 1 (LAMP1) further supports the hypothesis that ORF3a possibly hinders the interaction of lysosomes and autophagosomes.

**Figure 2:**
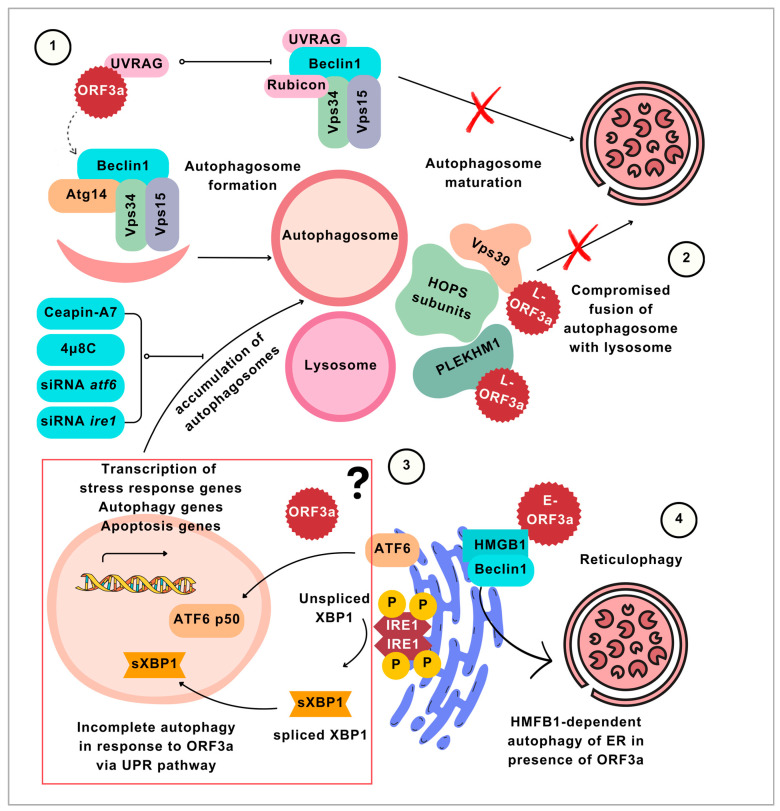
Three mechanisms (1–3) of incomplete autophagy stimulated by ORF3a and ORF3a-mediated reticulophagy (4). (1) ORF3a binds to UVRAG, which shifts the balance between two mutually exclusive Beclin1 complexes towards Beclin1-Atg14-Vps34-Vps16 (Beclin1–Atg14 complex) formation. The Beclin1–Atg14 complex stimulates the autophagosome formation. Meanwhile, the other complex of Beclin1 (Beclin1-UVRAG-Vps34-Vps15-Rubicon) is limited by the sequestering of UVRAG by ORF3a. The Beclin1–UVRAG complex is responsible for deriving the maturation of autophagosomes. Since the Beclin1–UVRAG complex is inhibited and more Beclin1 is available for forming the Beclin1–Atg14 complex, the process leads to more autophagosome formation and less autophagosome maturation; hence, incomplete autophagy is caused by ORF3a. (2) ORF3a inhibits HOPS complex formation by binding to HOPS complex subunits, thereby preventing the fusion of autophagosomes with lysosomes, leading to the accumulation of autophagosomes and incomplete autophagy. (3) ORF3a initiates incomplete autophagy through ATF6 and IRE1-mediated unfolded protein response (UPR). The crosstalk of autophagy and the UPR pathway has long been known; however, the mechanism by which SARS-CoV-2-ORF3a stimulates the pathway is not yet determined. The illustration of the ATF6 and IRE1 pathways and their relation to expressing autophagy genes is inspired by Estébanez et al. [62]. Downstream ATF6 and IRE1 pathways are ATF6 p50 and spliced XBP1, which translocate to the nucleus and may stimulate the autophagy genes in response to ER stress. To date, SARS-CoV-2 studies have not yet indicated which of the components of these pathways are directly influenced by ORF3a. However, it was determined that inhibiting the ATF6 and IRE1 through chemical inhibitors, Chapin-A7 and 4µ8C, and the gene knockdown of AFT6 or IRE1 reduce the autophagosome accumulation in ORF3a-transfected cells. (4) While the three mechanisms show the involvement of ORF3a in incomplete autophagy, ORF3a is also known to selectively stimulate the complete autophagy of the ER through HMGB1.

Among these viral autophagy regulators, SARS-CoV-2 ORF3a and ORF7a seem to play key roles in reprograming autophagy. Three potential mechanisms (Figure 2) by which ORF3a induces incomplete autophagy were proposed [37,41,48,49]: (i) interaction with UVRAG [41]; (ii) interaction with Vps39 [48,49,61]; and (iii) modulating the cross-talk between unfolded protein response (UPR) pathways and autophagy [37].

(i) Beclin1 forms two mutually exclusive complexes with Atg14 and UVRAG [63]. Atg14 facilitates the phosphorylation of Beclin1 at S30, which promotes autophagosome formation [64]. UVRAG complex drives autophagosomes maturation [65]. Qu et al. [41] presented the interaction of ORF3a with UVRAG, which resulted in decreased interaction of UVRAG with Beclin1. They also found increased interaction of Beclin1 with Atg14 in ORF3a-transfected cells [41], which was confirmed by another study [49]. Hence, this suggests that this imbalance between Atg14- and UVRAG-containing Beclin1 complexes was the potential cause of increased autophagosome formation and decreased autophagosome maturation [41]. However, there is also evidence of Atg14-mediated autophagosome maturation [66]. So, there could be alternative mechanisms of impaired autophagosome maturation in response to ORF3a. Moreover, the interaction of ORF3a with UVRAG might not necessarily be associated with dysregulation in autophagy. Some viruses, including influenza A virus and vesicular stomatitis virus, also exploit UVRAG for entry into the host cell [67].

(ii) Vps39 is a component of the HOPS complex that regulates autophagosome–lysosome fusion [17]. ORF3a was able to interact with Vps39 [48,49]. In normal conditions, the HOPS complex interacts with the GTPase Rab7 via Vps39 to promote autophagosome tethering on the lysosome. The binding of ORF3a prevents Vps39 from interacting with Rab7, thereby inhibiting the subsequent events of SNARE complex formation [48]. The mutated ORF3a could not bind Vps39 and its effects on autophagy were revoked [48]. Moreover, ORF3a prevents the interaction of Vps39 with Vps33A/Vps16 [49], the SNARE-binding subcomplex of the HOPS complex. As a result, the STX17–SNAP29–VAMP8 assembly is also compromised in the presence of ORF3a [48,49]. Expanding the previous knowledge of this mechanism underlying the autophagy blockage, a recent study [61] demonstrated the interaction of ORF3a with Vps39, which hindered its binding to PLEKHM1, an effector of Rab7 which is required for the tethering of vesicles on lysosomes.

(iii) Activating transcription factor 6 (ATF6), inositol-requiring enzyme (IRE), and protein kinase R-like ER kinase (PERK) are the three pathways of UPR activation [62]. Among these, the chemical inhibitors/gene knockdown of ATF6 or IRE prevents the ORF3a-induced increase in LC3-II levels [37]. This suggests that the crosstalk between UPR and the autophagy pathway [62] might be involved in ORF3a-induced perturbations in autophagy.

One of the later studies on SARS-CoV-2 [68] identified the role of ORF3a in brain pathology as a course of COVID-19. The angiotensin-converting enzyme 2 (ACE2), the functional receptor for viral entry, is profoundly expressed in neurons [69]. Hence, COVID-19 patients are susceptible to neurological damage [70]. The common neurological manifestations of COVID-19 are loss of olfaction and dizziness [71], while the rarely manifested severe cases include seizures and brain stroke [72]. The term “long-COVID” is associated with the long-lasting symptoms of COVID-19, including “brain fog”. Therefore, the understanding of the molecular basis of COVID-19-related neuropathology is crucial for efficient therapeutic approaches. Zhu et al. [68] demonstrated the molecular mechanism of ORF3a-derived neuropathies, which involves the autophagic pathway. The adeno-associated virus (AAV) delivery vector was employed to selectively express ORF3a in the brain of mice. As concluded by the previous studies, ORF3a blocks autophagic flux [37,48,49] in different cell line models. Likewise, ORF3a blocked autophagic flux in mouse brain, as shown by increased levels of LC3-II and p62 in ORF3a-expressing mice [68]. Consequently, the blocked autophagy leads to the accumulation of α-synuclein and glycosphingolipids [68], the macromolecules associated with neurodegeneration. These findings have clinical relevance for overcoming the neurological damage caused by COVID-19. In line with this study [68], a recent study [73] not only confirmed the autophagy blockage in ORF3a-transfected neuronal cells but also presented the prospective inhibitory effect of myelin basic protein (MBP) against ORF3a for restoring the autophagic flux. The colocalization and interaction of MBP with ORF3a suggest its role in counteracting ORF3a. The deimination of MBP could be one of the possible reasons for the worsened neurological manifestations of COVID-19 in multiple sclerosis patients [73].

As discussed earlier, ORF7a is another viral protein that has frequently been suggested as a regulator of the host cell’s autophagic pathway. Like ORF3a, ORF7a increases LC3-II levels [42,48,49]. The increase in autophagosome levels corresponds to the downregulation of the Akt/mTOR pathway [42], decreasing the mTOR-dependent inhibition of autophagy initiation components. Hence, ORF7a has the potential to induce autophagosome formation. However, it decreases the levels of the protein SNAP29 [42], a component of the SNARE complex, required for the fusion of autophagosomes with lysosomes [74]. The SARS-CoV-2 protein ORF7a reduces SNAP29 levels, presumably through caspase-dependent degradation via the activity of the caspase CASP3 [42]. The stimulation of vesicle formation and the inhibition of autophagic flux are potential viral mechanisms facilitating viral replication on the vesicles and shielding it from degradation.

Another mechanism by which ORF7a blocks the fusion of autophagosomes and lysosomes involves the interaction between ORF7a and p62 [75]. The interaction of ORF7a with p62 was first predicted computationally and then confirmed through a series of co-immunoprecipitation assays in HEK293T and A549 cell lines. The colocalization of LC3 and the lysosomal marker LAMP1 was decreased in ORF7a-transfected cells, which shows that ORF7a hinders the fusion of autophagosomes and lysosomes. The computationally predicted structure and physical properties of ORF7a could be important in developing strategies against this virulence protein. Through this computational modeling, glecaprevir, an anti-hepatitis C virus (HCV) drug, was predicted to be an inhibitor of ORF7a. Experimentally, glecaprevir exhibited potential against SARS-CoV-2 along with restoring autophagic flux, which presumably is the mechanism of clearing viral load by inhibiting ORF7a-mediated autophagy blockage [75].

The physiological functions of ORF3a and ORF7a have also been studied in yeast recently [76]. The expression of ORF3a and ORF7a resulted in the accumulation of p62 and Atg8, the homolog of mammalian LC3. Consistent with other studies [48,49], ORF3a led to the inhibition of autolysosome formation, assessed via the degradation of Atg8-GFP. The measure of free GFP was implemented to evaluate the digestion of Atg8-GFP. The cleavage of Atg8-GFP was decreased in the presence of ORF3a, while it was increased in the presence of ORF7a. These findings indicate a synergic function of ORF3a and ORF7a in autophagic degradation. The difference in the cellular localization of ORF3a (localized to the vacuole membrane) and ORF7a (localized with ER) [76] is also of relevance, as it might determine the effects of viral proteins on autophagy or selective autophagy. A recent study [77] provided a possible explanation of the distinct functions of viral proteins depending on their subcellular location by studying the two groups of ORF3a variants, which are localized with the endoplasmic reticulum (E-ORF3a) and lysosomes (L-ORF3a). Interestingly, L-ORF3a variants resulted in the accumulation of LC3-II in transfected cells, whereas E-ORF3a had little to no effect on LC3-II accumulation. One of the L-ORF3a variants, lacking the ability to bind Vps39, had a negligible effect on LC3-II accumulation, suggesting that the ability to block autophagy was compromised. Therefore, the studies discussed above suggest that SARS-CoV-2 proteins localized with lysosomes or vacuole membranes block autophagy by inhibiting the fusion of vacuoles with lysosomes. On the contrary, the viral proteins localized with the ER do not block autophagy [77] and rather facilitate reticulophagy [58,77], which will be discussed further in the next section.

ORF3a and ORF7a were also reported to block autophagy via other mechanisms [78]. ORF3a and ORF7a colocalized with endosomal Rab9 and exhibited two different methods of blocking autophagy. ORF3a decreased the colocalization of LC3 and lysosomes, while ORF7a blocked autophagy by reducing the acidity of lysosomes. In addition to ORF3a and ORF7a, NSP5 and NSP15 have been shown to modulate autophagy. NSP5 exhibits the potential to impair the p62-dependent autophagic degradation of ubiquitinated M-protein [79]. NSP5 expression does not affect the LC3-II levels. However, the NSP5-induced cleavage of p62 at the Q354 residue compromises its ability to bind LC3-II and ubiquitinated targets simultaneously [79], thus preventing the autophagic degradation of the target. This mechanism enables the virus to escape autophagic clearance. Another viral protein, NSP15, reduces the formation of autophagosomes, as indicated by lower LC3-II levels but increased p62 levels. The elevated p62 levels correspond to decreased autophagic activity, while the decreased LC3-II levels correspond to the reduced number of autophagosomes.

Apart from the respiratory and common neurological manifestations, COVID-19 is associated with altering spermatogenesis. In fact, testicular tissue expresses ACE2 [80], which makes it yet another target of viral pathogenesis. Kang et al. [59] performed a comprehensive analysis of the SARS-CoV-2 structural proteins E, M, N, and S in primary human sertoli cells. The sertoli cells form a blood–testis barrier (BTB), which is disrupted by autophagy induction. The transfection of sertoli cells with the SARS-CoV-2 structural proteins E, M, N, and S altered the expression of tight junction, adjacent junction, and gap junction proteins in a distinctive manner, suggesting the changes in BTB upon transfection. Among these proteins, E and M increased the number of degradative autophagic vacuoles (AVd) compared to the control vector. The structural proteins E and M increased the conversion of LC3-I to LC3-II while also increasing LC3-I levels, indicating enhanced autophagosome formation. The p62 levels were also increased, suggesting that the E- and M-induced autophagy is incomplete, as indicated by blockage of autophagic flux. The S and N proteins reduced LC3-II and increased the accumulation of p62, the combination showing less autophagy induction in response to S and N proteins. The treatment with autophagy inhibitor 3-MA rescued the effects of SARS-CoV-2’s structural proteins on the BTB-related proteins, which indicates that these proteins regulate the host’s BTB by hijacking autophagy.

### 2.3. The Interplay between SARS-CoV-2 and Selective Autophagy

Among the SARS-CoV-2 proteins that induce autophagy, ORF10 has a prominent role in selective autophagy [55]. ORF10 increased LC3-II levels and decreased p62 levels in HeLa cells. Moreover, there was an increased number of autolysosomes in ORF10-expressing cells. GFP-LC3 colocalized with mitophagy markers in ORF10-transfected cells. ORF10 reduced the levels of mitochondrial antiviral-signaling protein (MAVS). ORF10 was also found to interact with NIX, the autophagy receptor on the outer membrane of the mitochondria, and NIX-KO restored the levels of MAVS in ORF10-transfected cells [55]. Hence, the potential mechanism of ORF10-mediated mitophagy of MAVS is mediated by the interaction of ORF10 with NIX.

Unlike ORF10, NSP8 of SARS-CoV-2 induces rather ‘incomplete mitophagy’, characterized by increased levels of both LC3-II and p62 in transfected cells [47]. Like ORF3a and ORF7a, NSP8 leads to the accumulation of autophagosomes and blockage of autophagy in the A549 cell line stably expressing NSP8. Moreover, the transfection of Vero E6 and A549 with NSP8 increased the levels of LC3-II, which was also confirmed by quantifying LC3-EGFP puncta through fluorescence assays. This blockade of autophagic flux was also detected by the mCherry-EGFP-LC3 tandem reporter. NSP8-induced autophagy is associated with mitophagy, as indicated by the colocalization of LC3 with the mitochondrial marker TOM20 in NSP8-transfected cells. NSP8 leads to an increase in LC3-II levels in the mitochondrial fraction to a similar extent to the mitophagy inducer, carbonyl cyanide chlorophenylhydrazone (CCCP). The NSP8-induced mitophagy is incomplete autophagy, as the levels of mitochondrial proteins in NSP8-overexpressing cells were maintained, even with CCCP treatment, compared to control cells treated with CCCP, which showed reduced levels of mitochondrial proteins. This is further proven by the evidence from the Mito-DsRed2-EGFP tandem reporter. The potential mechanism underlying NSP8 inducing mitophagy is mediated by the damage caused by reactive oxygen species (ROS) to mitochondrial membranes, as the inhibition of ROS with the antioxidant NAC decreased autophagy induction in NSP8-transfected cells.

These findings of the above studies are in line with SARS-CoV-2-induced mitophagy [54], while nominating ORF10 and NSP8 as the viral factors involved in mitophagy. Contrarily, another study [57] showed decreased autophagy with ORF10 expression, indicated by the reduced LC3-II level. ORF10 colocalized and interacted with the adaptor protein, the stimulator of interferon genes (STING) [57]. STING has a role in autophagy induction [81]. The overexpression of STING restored the levels of LC3-II in ORF10-transfected cells [57]. Hence, the effects of ORF10 on autophagy are debatable. It can activate mitophagy [55] or decrease the non-canonical activation of autophagy by inhibiting STING [57]. MAVS and STING pathways regulate downstream signaling for IFN-β production [82]. The ORF10-mediated downregulation of MAVS and STING corresponds to suppressed IFN-β production [55,57].

Similarly, NSP13 of SARS-CoV-2 promotes the p62-dependent selective autophagy of TBK1 [56], a downstream signaling molecule in the MAVS cascade. The reduction in TBK1 was accompanied by suppression of IFN-β production [56]. The decrease in TBK1 levels was presumed to be autophagy-derived as the autophagy inhibitors restored the levels of TBK1 in the transfected cells. This study could not find a significant change in autophagy markers upon transfecting cells with NSP13 [56]. However, increased colocalization of TBK1 and LC3 was detected in NSP13-transfected HeLa cells [56]. Therefore, NSP13 potentially increases the recruitment of TBK1 to the autophagosomes instead of increasing autophagosome formation. The decreased levels of p62 and the colocalization of TBK1 with p62 in the presence of NSP13 provide further evidence of autophagy’s involvement in TBK1 degradation [56]. These findings were reproducible in SARS-CoV-2-infected Calu3 cells [56]. The levels of p62 and TBK1 in the infected cells decreased at 8 h post infection as a time-dependent variable. At later time points, the levels of p62 and TBK1 were recovered [56]. As explained earlier, the dynamic changes in autophagic activity upon SARS-CoV-2 infection might be associated with different viral components being expressed at different timepoints.

Another non-structural protein, NSP6, has recently shown the potential to degrade STING1 through ER-stress-induced autophagy [60]. Consequently, it decreases the phosphorylation levels of two downstream signaling molecules, IRF3 and TBK1, which are important molecules in IFN-β production. Moreover, the reduced levels of IFN-β1 in NSP6-transfected cells are in line with the downregulation of IRF3 and TBK1 [60]. Autophagic activity was detected in transfected cells with GFP-LC3 and mCherry-GFP-LC3. NSP6 transfection increased GFP-LC3 puncta, indicating an increase in autophagosome levels. The increased autophagosome levels were also observed with transmission electron microscopy. Moreover, NSP6 promoted the fusion of the autophagosomes with lysosomes, as shown by increased red puncta compared to green puncta in the mCherry-GFP-LC3 assay. Therefore, NSP6 induces complete autophagy, which is also demonstrated by increased LC3-II and decreased p62 levels at the protein level. The immunogold-labeled NSP6 localized on the ER and autophagosomes, as observed by immunoelectron microscopy [60], suggesting the involvement of the ER in NSP6-induced autophagy. Additionally, NSP6 increased the levels of heat shock protein family A (HSPA5) [60], which is released from the ER as a stress response. The ER stress inhibitor 4-phenylbutyric acid (4PBA) recovered the levels of STING1 in NSP6-transfected HEK293T cells [60]. Thus, ER stress mainly governs the NSP6-induced autophagy of SINTG1.

ORF3a of SARS-CoV-2 has dual functions in autophagy regulation. Multiple studies indicated the role of ORF3a in impairing autophagy [37,41,48,49]. Nevertheless, it also exhibits the potential to induce selective autophagy of the endoplasmic reticulum (ER) [58], referred to as reticulophagy. In A549 cells, ORF3a showed a tendency to increase autophagy, as indicated by the increased levels of LC3-II but the decreased levels of p62 [58]. ORF3a overexpression or SARS-CoV-2 infection significantly reduced the levels of the ER membrane proteins calnexin (CANX) and reticulon 4 (RTN4) and slightly increased the levels of the reticulophagy reporter RETREG1 [58]. Moreover, ORF3a-transfected cells had more autophagosomes and autolysosomes positive for the reticulophagy reporter SERP1/Ramp4 [58]. These findings suggest that ORF3a induces reticulophagy. ORF3a-induced reticulophagy stimulates the ER-stress-related genes [58]. The subsequent events in ORF3a-induced reticulophagy [58] and NSP6-induced autophagy of STING1 [60], and both illustrate the viral mechanism to counter host innate immunity. Hence, SARS-CoV-2 could benefit from the autophagy targets that regulate host innate immunity via IFN-β production.

In summary, SARS-CoV-2 infection leads to an overall higher turnover of autophagosomes and incomplete autophagy [38,40,41,42,43,44,45,46,48,49]. The key viral components that cause impaired autophagy are ORF3a [37,41,48,49] and ORF7a [42,48,49]. The blocked autophagy prevents viral clearance and the accumulation of autophagosomes potentially facilitates viral replication. While the intact virus decreases autophagic flux [38,40,41,42,43,44,45,46,48,49], some viral proteins have a synergic effect on autophagy, facilitating the selective autophagy of STING [60], TBK1 [56], the mitochondria [47,55], and the ER [58]. These targets of selective autophagy converge on pathways leading to IFN-β production. Thus, ORF10, NSP6, NSP8, and NSP13 selectively induce the autophagy of host components responsible for defense mechanisms. Interestingly, ORF3a protein has a dual role in autophagy, as it blocks the autophagic flux in multiple cell lines [37,41,48,49] but also promotes the autophagy of the ER [58] in A549 cells. These different roles could be caused by the phases of the viral life cycle in the host cells. As discussed earlier, the levels of target proteins and autophagy markers fluctuate at different time points [56]. One of the possible reasons for this is different viral proteins with distinct roles peak at different time points after infection. Another explanation is the evolution of the variant forms of viral proteins, which may alter their intracellular location and function. For example, the study of ORF3a variants elucidated different roles of ER-localized ORF3 (E-ORF3a) and lysosomal-localized ORF3 (L-ORF3a) [77].

## 3. Effects of Autophagy on SARS-CoV-2 Replication

The studies discussed so far demonstrated that SARS-CoV-2 and its proteins significantly affect autophagy. Hence, another question arises: do these autophagy perturbations favor SARS-CoV-2, or do they occur as a host response to the infection?

Gassen et al. [38] demonstrated that the replication of SARS-CoV-2 was inhibited in VeroFM and Calu-3 cells upon treatment with compounds that enhance autophagic activity, and these compounds are SMIP004 (Beclin1 stabilizers), valinomycin, and niclosamide, which promote autophagy initiation, as well as rapamycin and MK2206, which increase autophagy through downregulation of the mTOR/AKT pathway. Moreover, the compounds spermidine and spermine, stabilizers of Vps34, also had a negative effect on SARS-CoV-2 infection.

On the other hand, the inhibitors of autophagy, targeting ULK1 and Vps34, increased the genome equivalent (GE) of SARS-CoV-2 in the cells. This study concluded that autophagy stabilizers reduce viral replication, whereas autophagy inhibitors facilitate viral propagation (Table 1). In contrast to this study [38], Vps34 inhibitors exhibited the potential to reduce SARS-CoV-2 propagation in Calu-3 cells [83]. Similarly, the autophagy inhibitor 3-methyladenine (3-MA) decreased viral titers in SARS-CoV-2-infected Caco2 and Vero E6 cells [42]. However, the findings in these studies cannot be directly associated with autophagy, as the target proteins, ULK1, Beclin1, mTOR, AKT, and Vps34, have a broad range of functions besides regulating autophagy. Likewise, the inhibitor 3-MA is not specific to autophagy and might affect the apoptotic pathway as well.

The impact of autophagy on SARS-CoV-2 was also evaluated through gene knockout and knockdown methods. Downregulating autophagy in hACE2-expressing MEF cells with Atg3 or Atg5 knockout significantly decreased SARS-CoV-2 transcripts [41]. Moreover, siRNA knockdown of Atg7 in HeLa cells significantly decreased N protein levels and viral titers [42]. These results suggest a pro-viral role of autophagy initiation in SARS-CoV-2 replication. However, over-expressing SNAP29 reduces viral load in the infected cells [42]. These results show that the initial phases of autophagy might favor viral production, whereas complete autophagy leads to viral clearance. The over-expression of SNAP29 and caspase-resistant mutant SNAP29 overcomes the autophagy blockage, which is conferred by ORF7a via caspase-dependent SNAP29 degradation [42]. Notably, in Huh7-Lunet/T7 cells, Atg5 KO and Atg16L KO did not affect SARS-CoV-2 replication, determined by qRT-PCR and N protein expression [84], which may suggest distinct roles of autophagy in different cell lines.

## 4. Pharmacological Strategies to Fight SARS-CoV-2 by Moderating Autophagy

There was and continues to be a desperate need for effective antivirals against SARS-CoV-2 infection, especially at the early stage of the COVID-19 pandemic, which motivated researchers to screen several approved drugs for repurposing them in the treatment of COVID-19. In the early attempts to investigate the mechanisms of potent drugs for COVID-19, the activity profiles of the drugs and their potential to reduce cytopathic effect (CPE) were compared [85]. The data revealed a positive correlation between the anti-autophagic activity of the drugs and their anti-CPE activity [85].

As indicated by several studies reviewed in this article, SARS-CoV-2 initiates the autophagosome formation, blocks the autophagic flux, and compromises the interferon production by means of mitophagy and downregulating the non-canonical STING-GAS pathway of autophagy. Thus, the therapeutic strategies include restoring the autophagic flux [75,86] or contrarily blocking the autophagic flux to prevent viral egress [87,88]. Another possibility could be to prevent the autophagic targeting of proteins that promote host immune responses, i.e., MAVS and STING.

Theoretically, restricting the initial vesicle formation could also limit the viral propagation by reducing autophagosomes, which SARS-CoV-2 hijacks for its replication. However, inhibiting the initiation complex components, e.g., Vps34 and ULK1, has varying effects on SARS-CoV-2 in vitro [38,83]. Similarly, Beclin1 stabilizers or mTOR/Akt inhibitors that promote autophagy initiation reduce SARS-CoV-2 propagation in cell models [38]. It is also important to consider that these protein targets are multifunctional. Hence, inhibition and stability are not only associated with autophagy but also with other cellular functions.

The anti-HCV drug glecaprevir was suggested as a therapeutic strategy against SARS-CoV-2 [75]. This drug was screened by predicting drug-binding pockets of SARS-CoV-2 ORF7a protein, followed by experimental validation. Glecaprevir reduces the number of replication-competent SARS-CoV-2-GFP/ΔN particles (N gene replaced with GFP) in Caco-2-N and Caco-2 cell lines. Glecaprevir restored the autophagic flux, as the accumulation of p62 decreased in infected cells pretreated with glecaprevir. However, the number of replication-competent SARS-CoV-2-GFP/ΔN/ΔORF7a (particles lacking ORF7a) was not affected by glecaprevir pre-treatment. Thus, the results indicate that glecaprevir might be a competitive inhibitor of ORF7a, as the pretreatment only inhibited the infection with viral particles containing ORF7a (SARS-CoV-2-GFP/ΔN). One of the mechanisms by which the virulence protein ORF7a blocks autophagic flux is interacting with p62. Glecaprevir treatment re-establishes the colocalization of p62 with lysosomal LAMP1 in ORF7a-transfected cells. Thus, glecaprevir potentially reduces the infection by restoring autophagic flux in the infected cells, which can lead to viral clearance. Likewise, another drug, olmesartan, also promotes autophagic flux specifically for TGF-β to overcome the SARS-CoV-2-E-protein-induced renal fibrosis [86]. The autophagic degradation of TGF-β reduces inflammation, which drives renal fibrosis.

Inducing lipophagy using niclosamide [89,90] is another example of restoring autophagy to control lipid metabolism, which is affected by SARS-CoV-2 infection [89]. Garrett et al. [89] demonstrated that niclosamide decreases certain plasmogens in SARS-CoV-2-infected Vero E6 cells, which are otherwise elevated with SARS-CoV-2 infection without treatment. The over-representation of autophagy genes in Gene Ontology (GO) terms indicates the stimulation of autophagy by niclosamide treatment in SARS-CoV-2-infected cells. The control of lipid metabolism with niclosamide treatment suppresses viral infection, presumably by lipophagy, a form of autophagy that engulfs cellular lipid droplets of unsaturated triglycerides that may aid viral entry and trafficking.

While several studies demonstrate the SARS-CoV-2-dependent blockage of autophagy, it is interesting that initially, the drugs that also block autophagic flux were implicated in COVID-19 [40,44,91,92,93]. The working mechanisms of these drugs are mediated by the alkalization of lysosomes or by the inhibition of late-stage autophagy, thereby preventing viral entry or egress. Among those lysosome inhibitors, hydroxychloroquine (HCQ), an off-patent antimalarial drug also used to treat autoimmune diseases, was suggested as a good candidate for COVID-9 treatment based on the significant inhibitory effects on SARS-CoV-2 in vitro [40]. Unfortunately, clinical trials showed that HCQ did not have clinical benefit for COVID-19 [94,95,96]. The reason for this is ascribed to the two distinct SARS-CoV-2 cell entry pathways: endosomal entry (clathrin-mediated endocytosis) and cell surface entry (membrane fusion) [97]. The uncoating of SARS-CoV-2 RNA to the cell cytoplasm in an endosomal entry manner is mediated by the lysosome, while it is triggered by cell membrane fusion in the cell surface entry way. SARS-CoV-2’s entry into Vero cells occurs through an endosomal entry route, and the deregulation of proteases in lysosomes by HCQ or other lysosome inhibitors will markedly suppress SARS-CoV-2 replication. However, in the real scenario of infection in humans, entry of SARS-CoV-2 into upper respiratory epithelia cells and especially into lung cells mainly occurs through the cell surface fusion pathway [97]. Chloroquine exhibited no inhibitory effects on SARS-CoV-2 infection in human lung cells [98].

Cloherty et al. [88] proposed the potential of berbamine dihydrochloride (BBM) and daurisoline (DAS) to limit SARS-CoV-2 Omicron infection in Caco2 cells and epithelial monolayer models by blocking autophagic flux. It is worth noting that SARS-CoV-2 Omicron can utilize both the endosomal and cell surface fusion entry routes but prefers endosomal fusion to cell surface fusion when entering cells. Caco2 cells express TEMPRSS2 (required for cell surface fusion entry) and cathepsin L (required for endosomal entry) [99,100]. BBM or DAS stabilized the cell–cell contacts and epithelial integrity of the infected epithelial monolayer. BBM and DAS treatment blocked autophagic flux, which was determined by p62 accumulation. BBM and DAS are not classic autophagy-regulating drugs. Therefore, the mode of action for BBM was further studied by knocking down Bcl-2 interacting protein 3 (BNIP3), a molecule that inhibits autophagosome–lysosome fusion. In Caco2 cells with knockdown of BNIP3, the preventive effect of BBM against SARS-CoV-2 was canceled out. Hence, BBM restricts SARS-CoV-2 through the BNIP3-dependent blockage of autophagic flux [88]. The therapeutic effects of BBM and DAS might also be attributed to the prevention of viral egress through the endosomal pathway.

In addition to the drugs targeting autophagy, utilizing the autophagic machinery against the viral component could be a strategy for designing next-generation vaccines. One such approach was proposed by Wen et al. [101], where they demonstrated the use of the fusion protein N-LC3b to enhance T-cell functionality against N-antigen. The fusion protein of SARS-CoV-2 N and mouse LC3b colocalized with lysosomal markers and MHC-II in the transfected cells. The fusion protein in the mice model resulted in increased production of IFN-γ and TNF-⍺ by CD8+ T cells.

## 5. Summary and Perspective

Viruses, especially emerging viruses, and their associated diseases pose long-standing threats to the human population. The development of effective antiviral treatments is urgently needed for the next outbreak or pandemic. Autophagy is a promising target that can be modulated to ameliorate virus-related diseases. Based on the current literature, autophagy plays multifaceted roles in SARS-CoV-2 infection. In relation to virus infection, SARS-CoV-2 induces autophagy to promote viral replication while blocking the selective autophagy targeting viral proteins and hijacks autophagosome-derived secretory organelles to release the progeny virus from the host cells. Autophagy is closely engaged in the whole life cycle of SARS-CoV-2 infection and exhibits distinct roles at different stages of viral infection. Therefore, further studies are needed to clarify the comprehensive scenario of virus–autophagy interaction in the viral lifecycle and determine the key factors essential for SARS-CoV-2 or other pathogenic coronavirus infections.

Additionally, as a highly conserved degradation mechanism in all eukaryotic cells, autophagy has complicated connections with other biological processes, such as inflammatory reactions. In addition to the direct damage caused by the virus, the virus-induced hyperreactive inflammation, also called the ‘cytokine storm’, is the main player driving severe disease or even leading to death in viral diseases. It has been reported that the immunomodulatory drug niclosamide, an autophagy inducer, is able to inhibit SARS-CoV-2 replication [38,89] and impede SARS-CoV-2-induced inflammation [102]. Prospective studies of pharmaceutical activation of autophagy against SARS-CoV-2 infection indeed provided promising results [103,104], which suggests that moderating autophagy could have a ‘one stone and two birds strategy’ against SARS-CoV-2-associated diseases.

## Figures and Tables

**Table 1:** Effect of autophagy-regulating compounds/genes on SARS-CoV-2 growth.

Treatment/ Knockout Method	Cell Line/ Model	Function/ Mode of Action	Effect on SARS-CoV-2	Reference
**SMIP004**	VeroFM and Calu-3	Beclin1 stabilizer	Less PFU and GE	[38]
**Valinomycin**	VeroFM and Calu-3	Beclin1 stabilizer	Less PFU and GE	[38]
**Niclosamide**	VeroFM and Calu-3	Beclin1 stabilizer	Less PFU and GE	[38]
**Rapamycin**	VeroFM	mTOR inhibitor	Less PFU and Increased GE	[38]
**MK-2206**	VeroFM and Calu-3	Akt inhibitor	Less PFU and GE	[38]
**Spermidine (spd)**	VeroFM and Calu-3	Downregulates EP300 (inhibitor of Vps34)	Less PFU and GE	[38]
**Spermine (spm)**	VeroFM and Calu-3	Downregulates EP300 (inhibitor of Vps34)	Less PFU and GE	[38]
**AICAR**	VeroFM	Inhibitor of AMPK	No significant effect	[38]
**MRT68921**	VeroFM	Inhibitor of ULK1	Increased GE	[38]
**SAR405**	VeroFM and Calu-3	Inhibitor of Vps34	Increased GE in VeroFM [38], reduced viral titer in Calu-3 [83]	[38,83]
**VPS34-IN1**	Vero E6 and Calu-3	Inhibitor of Vps34	Reduced SARS-CoV-2-induced CPE.	[83]
**PIK-III**	Vero E6 and Calu-3	Inhibitor of Vps34	Reduced SARS-CoV-2-induced CPE	[83]
**Triacsin C**	Vero E6 and Calu-3	Inhibitor of Vps34	Reduced SARS-CoV-2-induced CPE	[83]
**Orlistat**	Vero E6 and Calu-3	Inhibitor of Vps34	Reduced SARS-CoV-2-induced CPE	[83]
**3-MA**	Caco2 and Vero E6	Downregulate autophagy	Decreased viral titre	[42]
**Atg3/Atg5 knockout**	MEF	Compromised autophagy machinery	Decreased viral transcripts	[41]
**siRNA knockdown of Atg7**	HeLa cells	Compromised autophagy machinery	Decreased N-protein and viral titer	[42]
**Atg5/Atg16L knockout**	Huh7-Lunet/T7	Compromised autophagy machinery	No significant change in viral transcripts	[84]
